# Cyanorak v2.1: a scalable information system dedicated to the visualization and expert curation of marine and brackish picocyanobacteria genomes

**DOI:** 10.1093/nar/gkaa958

**Published:** 2020-10-30

**Authors:** Laurence Garczarek, Ulysse Guyet, Hugo Doré, Gregory K Farrant, Mark Hoebeke, Loraine Brillet-Guéguen, Antoine Bisch, Mathilde Ferrieux, Jukka Siltanen, Erwan Corre, Gildas Le Corguillé, Morgane Ratin, Frances D Pitt, Martin Ostrowski, Maël Conan, Anne Siegel, Karine Labadie, Jean-Marc Aury, Patrick Wincker, David J Scanlan, Frédéric Partensky

**Affiliations:** Sorbonne Université & CNRS, UMR 7144 ‘Adaptation & Diversity in the Marine Environment’ (AD2M), Station Biologique de Roscoff (SBR), 29680 Roscoff, France; Sorbonne Université & CNRS, UMR 7144 ‘Adaptation & Diversity in the Marine Environment’ (AD2M), Station Biologique de Roscoff (SBR), 29680 Roscoff, France; Sorbonne Université & CNRS, UMR 7144 ‘Adaptation & Diversity in the Marine Environment’ (AD2M), Station Biologique de Roscoff (SBR), 29680 Roscoff, France; Sorbonne Université & CNRS, UMR 7144 ‘Adaptation & Diversity in the Marine Environment’ (AD2M), Station Biologique de Roscoff (SBR), 29680 Roscoff, France; CNRS & Sorbonne Université, FR 2424, ABiMS Platform, Station Biologique de Roscoff (SBR), F-29680 Roscoff, France; CNRS & Sorbonne Université, FR 2424, ABiMS Platform, Station Biologique de Roscoff (SBR), F-29680 Roscoff, France; CNRS & Sorbonne Université, FR 2424, ABiMS Platform, Station Biologique de Roscoff (SBR), F-29680 Roscoff, France; Sorbonne Université & CNRS, UMR 8227 ‘Integrative Biology of Marine Models’ (LBI2M), Station Biologique de Roscoff (SBR), F-29680 Roscoff, France; Sorbonne Université & CNRS, UMR 7144 ‘Adaptation & Diversity in the Marine Environment’ (AD2M), Station Biologique de Roscoff (SBR), 29680 Roscoff, France; CNRS & Sorbonne Université, FR 2424, ABiMS Platform, Station Biologique de Roscoff (SBR), F-29680 Roscoff, France; Sorbonne Université & CNRS, UMR 7144 ‘Adaptation & Diversity in the Marine Environment’ (AD2M), Station Biologique de Roscoff (SBR), 29680 Roscoff, France; CNRS & Sorbonne Université, FR 2424, ABiMS Platform, Station Biologique de Roscoff (SBR), F-29680 Roscoff, France; CNRS & Sorbonne Université, FR 2424, ABiMS Platform, Station Biologique de Roscoff (SBR), F-29680 Roscoff, France; CNRS & Sorbonne Université, FR 2424, ABiMS Platform, Station Biologique de Roscoff (SBR), F-29680 Roscoff, France; Sorbonne Université & CNRS, UMR 7144 ‘Adaptation & Diversity in the Marine Environment’ (AD2M), Station Biologique de Roscoff (SBR), 29680 Roscoff, France; University of Warwick, School of Life Sciences, Coventry CV4 7AL, UK; University of Warwick, School of Life Sciences, Coventry CV4 7AL, UK; Université de Rennes 1, INSERM, EHESP, IRSET, F-35043 Rennes, France; Université de Rennes 1, INRIA, CNRS, IRISA, F-35000 Rennes, France; Genoscope, Institut de biologie François-Jacob, Commissariat à l’Energie Atomique (CEA), Université Paris-Saclay, F-91000 Evry, France; Genoscope, Institut de biologie François-Jacob, Commissariat à l’Energie Atomique (CEA), Université Paris-Saclay, F-91000 Evry, France; Génomique Métabolique, Genoscope, Institut de biologie François Jacob, CEA, CNRS, Université d’Évry, Université Paris-Saclay, F-91000 Evry, France; University of Warwick, School of Life Sciences, Coventry CV4 7AL, UK; Sorbonne Université & CNRS, UMR 7144 ‘Adaptation & Diversity in the Marine Environment’ (AD2M), Station Biologique de Roscoff (SBR), 29680 Roscoff, France

## Abstract

Cyanorak v2.1 (http://www.sb-roscoff.fr/cyanorak) is an information system dedicated to visualizing, comparing and curating the genomes of *Prochlorococcus*, *Synechococcus* and *Cyanobium*, the most abundant photosynthetic microorganisms on Earth. The database encompasses sequences from 97 genomes, covering most of the wide genetic diversity known so far within these groups, and which were split into 25,834 clusters of likely orthologous groups (CLOGs). The user interface gives access to genomic characteristics, accession numbers as well as an interactive map showing strain isolation sites. The main entry to the database is through search for a term (gene name, product, etc.), resulting in a list of CLOGs and individual genes. Each CLOG benefits from a rich functional annotation including EggNOG, EC/K numbers, GO terms, TIGR Roles, custom-designed Cyanorak Roles as well as several protein motif predictions. Cyanorak also displays a phyletic profile, indicating the genotype and pigment type for each CLOG, and a genome viewer (Jbrowse) to visualize additional data on each genome such as predicted operons, genomic islands or transcriptomic data, when available. This information system also includes a BLAST search tool, comparative genomic context as well as various data export options. Altogether, Cyanorak v2.1 constitutes an invaluable, scalable tool for comparative genomics of ecologically relevant marine microorganisms.

## INTRODUCTION

The regular decrease in sequencing costs associated with the rapid development of Next Genome Sequencing (NGS) technologies has led to the multiplication of microbial genomes (1,2), making possible extensive comparative genomics studies. Genomes are generally annotated using gene calling programs, such as e.g. RAST (3) or Prokka (4), which can provide fairly reliable annotations for the most conserved core genes involved in general metabolism (e.g. ribosomal proteins, Krebs or Calvin cycle enzymes, DNA replication, tRNA, etc.) or more specific but well characterized functions shared by many sequenced organisms (chlorophyll biosynthesis, nitrogen fixation, etc.). However, these automatic annotations are much less reliable for the least conserved accessory genes, such as those encoding enzymes responsible for cell wall biosynthesis that are often multi-domain, with highly variable domain composition, or those coding for species- or even strain-specific functions (e.g. biosynthesis of secondary metabolites, carotenoids, etc.). Thus, even though an initial step of automatic annotation is mandatory, functional annotation of predicted coding sequences (CDS) still requires extensive expert human curation to be reliable, especially for non-model organisms. With the exponential increase of newly sequenced genomes, manually curating individual genomes is however a highly time-consuming and inefficient approach. A smart alternative is to curate several phylogenetically related genomes at a time, after gathering sequences into Clusters of Likely Orthologous Genes (CLOGs), i.e. genes that exhibit reciprocal best hits to one another and are hypothesized to have the same function in the different members of the dataset (5,6). This strategy, notably used in the NCBI’s ‘prokaryotic genome annotation pipeline’ (7) for annotating new genomes or re-annotating older genomes before inclusion in the RefSeq database, allows propagating rich, functional annotations made at CLOG level to all proteins composing the CLOG and makes it possible to unify and standardize these annotations across all sequenced strains.

Here we present Cyanorak v2.1, an information system based on CLOGs that is dedicated to the annotation, comparison and visualization of picocyanobacterial genomes. Initially created in the mid-2000s by A. Dufresne and co-authors to compare the first 14 genomes of marine and brackish picocyanobacteria (8), the Cyanorak database has significantly increased since then and relies on a completely redesigned and tremendously enriched information system (v2.1) which, contrary to Cyanorak v1, is scalable, i.e. conceived to allow addition of more genomes. The current database encompasses 95 genomes and two metagenome-assembled genomes (MAGs), including 31 newly released *Synechococcus* and *Cyanobium* genomes (9), which have been closed using a custom-designed assembly and scaffolding pipeline (10). All strains whose genomes have been included in the Cyanorak v2.1 database belong to Cyanobacteria Subsection I, Cluster 5 *sensu* Herdman (11), a short-rod shaped group that forms a deep monophyletic branch within this ancient phylum (12). The common ancestor of all Cluster 5 members is thought to have diverged from other cyanobacteria about 1 Gy ago, during the Mesoproterozoic period (13). Members of Cluster 5 are also called ‘α-cyanobacteria’, based on the occurrence in their cytoplasm of specific α-type carboxysomes, phylogenetically and structurally closer to that of thiobacilli than to the β-type carboxysomes found in all other cyanobacteria, so-called ‘β−cyanobacteria’ (14). Cluster 5 itself is split into four major groups, including the monophyletic, strictly marine *Prochlorococcus* lineage and three deeply branching groups, called sub-clusters (SC) 5.1 through 5.3 (8,15,16). Based on the comparison of 81 non-redundant genomes, Doré and coworkers recently suggested to rename them *Ca*. Marinosynechococcus (SC 5.1), *Cyanobium* (SC 5.2) and *Ca*. Juxtasynechococcus (SC 5.3) (9). SC 5.1 is the most diversified of all these lineages, with about 10 phylogenetically distinct clades based on 16S rDNA phylogeny (16) and 11 to 16 using higher resolution markers (17,18). Members of these clades are all strictly marine except clade VIII that specifically gathers halotolerant strains. SC 5.2 also mostly encompasses halotolerant strains as well as one freshwater representative (*Cyanobium gracile* PCC 6307). While members of SC 5.3 were initially thought to be strictly marine (8,16), freshwater members of this group were recently discovered in various lakes (19,20). The current version of Cyanorak v2.1 encompasses representatives of most of the lineages (SC and clades) known to date in Cluster 5, with the exception of the newly described freshwater members of SC 5.3 as well as members of the yet-uncultured SC 5.1 clades EnvA and EnvB (18,21). Since all Cluster 5 members possess a similar morphology (spherical to rod shaped) and lifestyle (aquatic, non-diazotrophic oxyphototrophs; (11)) and form a monophyletic branch within the Cyanobacteria phylum, we assume that members of most CLOGs defined within this genetically homogeneous group exhibit the same function, though this may not be true when considering more distant organisms, notably cyanobacteria from other clusters that exhibit different lifestyles. Here, we describe the construction of the Cyanorak v2.1 database, the rich functional annotation available for each CLOG and the tools and plugins that were developed to explore the genomic diversity of this ecologically relevant group of organisms, which has recently become one of the main microbial models in marine ecology.

## MATERIALS AND METHODS

### Clustering of likely orthologous sequences and CLOG curation

**Figure 1.**
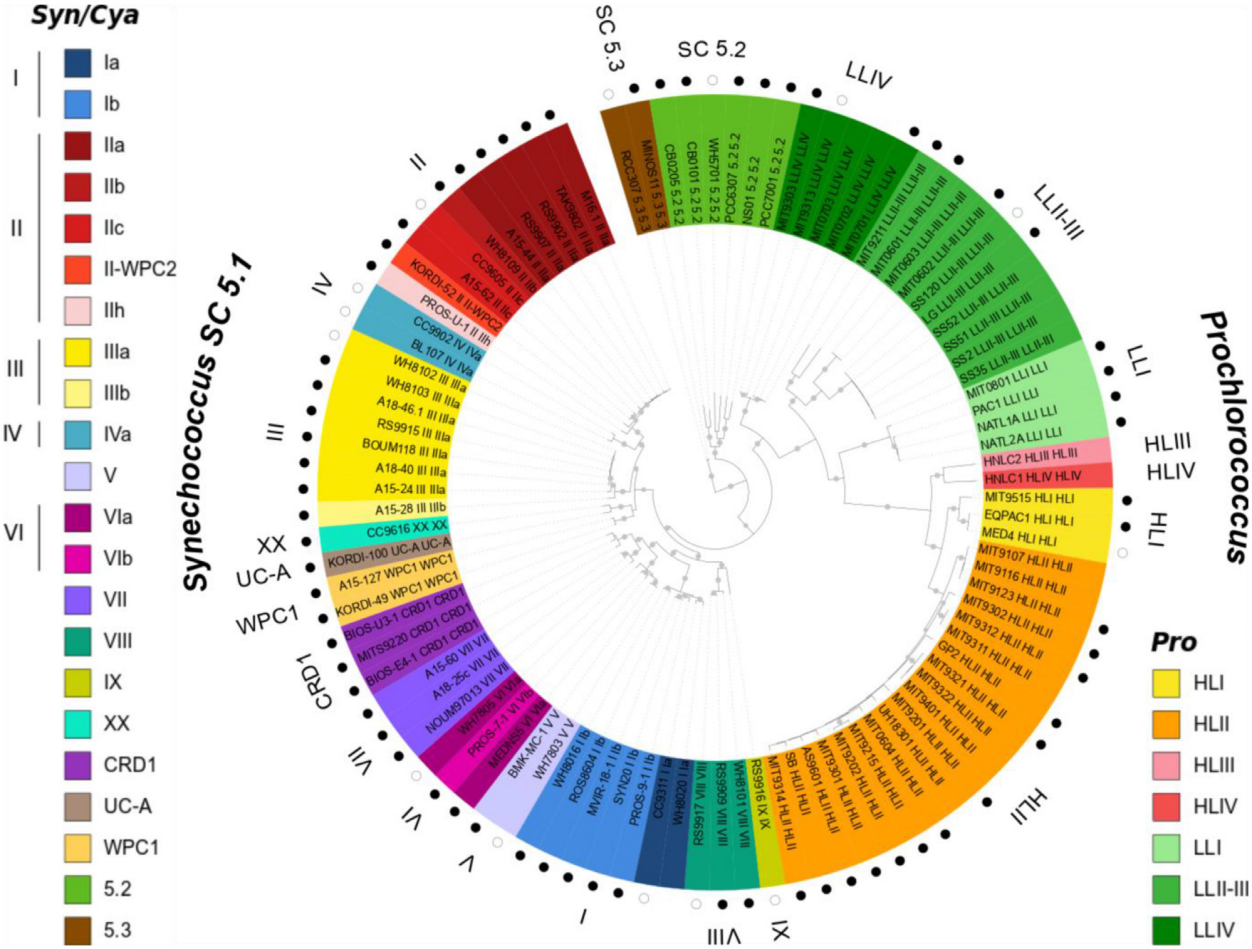
Maximum likelihood tree based on 579 core proteins showing the phylogenetic relatedness of the 97 genomes of the current Cyanorak v2.1 database. Grey dots indicate bootstrap support over 70%. Sequences were named after strain name followed by clade and subclade when available (sub-clade assignments as in 21) and the background colors correspond to the finest possible taxonomic resolution obtained for each strain using the *petB* marker gene (left hand side legend for *Synechococcus*, right hand side legend for *Prochlorococcus*). The 81 non-redundant, high quality genomes used by Doré *et al.* (9) for comparative genomics are indicated by a circle surrounding the tree and among them the 14 first genomes used in the previous version of Cyanorak (v1) are shown as empty circles.

Following the construction of a first series of CLOGs based on the 14 first sequenced picocyanobacterial genomes (8), Cyanorak v1 CLOG numbers have been cited in a number of publications from our group (see, e.g. (22–30)). In order to preserve at best these preexisting CLOG numbers after the addition of 83 new genomes either retrieved from Genbank or newly sequenced at our initiative (Figure 1; (9)), all CDS from the 97 genomes were first clustered using all-against-all BLASTP+ comparison (31) and the OrthoMCL clustering algorithm (32) with an e-value threshold of 10^−5^ and new CLOGs were then mapped to previously defined Cyanorak v1 CLOGs. New CLOGs containing all sequences from a v1 CLOG plus additional sequences from new genomes as well as manually curated CLOGs were assigned the previous v1 CLOG numbers. All other sequences were then tentatively assigned to preexisting CLOGs using HMMER (33) with an e-value threshold of 10^−20^ and remaining sequences were finally clustered using OrthoMCL to define new CLOGs or left as singletons in individual CLOGs if not clustered.

While Cyanorak v1 contained only CDS, these steps also allowed us to generate CLOGs for rRNAs, tRNAs, tmRNAs using all-against-all BLASTN+ and the OrthoMCL algorithm using the same threshold as for CDS (32). After this semi-automatic clustering step, a large number of CLOGs (∼4300, i.e. 17% of all CLOGs; Supplementary Figure S1) were further manually curated in order to (i) check and complement the functional description of CLOGs and (ii) verify that members of a given CLOG were truly orthologs, based on their phyletic pattern, alignments and phylogenetic trees. Paralogs were moved into different CLOGs when they grouped together into different branches as the *bona fide* orthologs. In order to refine assessment of the core genome (9), >1750 genes missed by gene prediction software tools, either because they were too short (e.g. *petM*, *psbM*) or partially overlapping with other genes notably in the case of long 3′-3′ overlaps (e.g. for *pyrB-ndbA or panB-hemN*), were also manually added to different genomes. Furthermore, many over-predictions of ORFs (e.g. short CDS of unknown function totally overlapping long annotated CDS) were eliminated, and this even from genomes retrieved from Genbank. Finally, many start positions were corrected from obviously too short or too long sequences, based on an alignment of all CLOG members and/or 5′-end extensions using TBLASTN searches.

### Implementation of the Cyanorak v2.1 information system

The development of Cyanorak v2.1 was done in two steps. The first version (v2.0) of this information system included a history feature to keep track of every change and allowing to readily revert any change at a very granular level as well as enabling curators to check the journal of changes undergone by every gene or CLOG. This private version of the information system is still currently used for the manual curation of the database. However, in order to give the general public access to the curated data with the best possible response times, especially now that the number of genomes and MAG’s in the database has risen to 97, a completely new version (v2.1) of the Cyanorak information system, devoid of the history feature, was recently developed and proved to be two to three times faster than v2.0. Two instances of the Cyanorak information system therefore co-exist on our server: (i) the restricted access, editable Cyanorak v2.0 version allowing expert curators to edit most fields of the ‘CLOG’ and ‘gene’ pages and (ii) the publicly accessible, non-editable Cyanorak v2.1 version, the latter corresponding to the state of the Cyanorak database at the time of publication of a comparative genomics study of the 81 non-redundant, high quality genomes of the database (9) and of an extensive transcriptomic analysis of the response of the *Synechococcus* sp. WH7803 strain to various environmental stresses (34). This public version will be regularly updated in the future, with concomitant changes in version number, when new whole genome sequences (WGS), single amplified- and metagenome assembled- genomes (SAGs, MAGs) and/or transcriptomes either retrieved from public databases (e.g. Genbank) or generated by our group will be added to the Cyanorak database and described in the frame of forthcoming publications. A restricted access, editable instance based on the v2.1 implementation is currently being developed and should replace the current v2.0 instance in the near future for expert curation purposes.

On a technical level, the bulk of Cyanorak v2.1 has been implemented using the Java programming language, with an extensive use of the Spring framework. The data itself is stored in a relational database (PostgreSQL), and the link between the application and the database is done through an object relational mapper (Hibernate). A small set of Python auxiliary tools has also been developed, mostly to prepare the data for import, to post-process exported data or to perform specific batch updates.

## RESULTS

### General characteristics of the database

Built from 97 picocyanobacterial genomes, including 43 *Prochlorococcus* and 54 *Synechococcus*/*Cyanobium*, which are representative of the wide genetic and pigment diversity existing within these genera (Figure 1), Cyanorak v2.1 encompasses 252,176 genes that were split into 25,834 CLOGs. A plot of the distribution of the number of sequences per CLOG expectedly shows that the most frequent categories are CLOGs with one sequence, i.e. unique genes (15,283 CLOGs), and CLOGs with few (2-5) members (Supplementary Figure S1). Although most of these CLOGs (e.g. 91% of unique genes) are annotated as ‘hypothetical’ or ‘conserved hypothetical’ proteins, a number of them display a more precise functional annotation, since they share some similarities to genes or domains of known function, with among the most abundant: glycosyltransferases, restriction-modification system proteins, integrases, transposases, methyltransferases, NAD-dependent epimerases/dehydratases and tetratricopeptide repeat (TPR) family proteins. The next most abundant CLOG category (611 CLOGs) is the one containing 97 sequences, which corresponds to the picocyanobacterial core genome *sensu stricto*. As expected, this number is significantly lower than the picocyanobacterial strict core gene set (911 genes) estimated by Doré *et al.* (9) using the 81 non-redundant, high-quality genomes of the Cyanorak database. Yet, given that some of the 97 genomes or MAG’s, especially those not included in this 81-genome set, are incomplete and/or contain frameshifted genes (in this case, the two or more gene fragments resulting from a frameshift have been put into the same CLOG), many CLOGs contain a number of genes that is close but not exactly equal to 97. So, the picocyanobacterial core genome *sensu lato* is likely much larger than 611 genes, and we estimated it using a relaxed definition of core genes (CLOG is considered as core of a taxonomic group if it is present in ≥90% of the strains within this group) to be 1,271 genes. A small number of CLOGs contain a large number of sequences, i.e. between 105 and 337 sequences. These CLOGs most often contain paralogous sequences that cannot be split into different CLOGs based on phylogenetic analyses. These notably include the identical multi-copy genes encoding the photosystem II core proteins D1.2 and D2, porins, AbrB-like transcriptional regulators and the high-affinity phosphate-binding protein PstS.

### Cyanorak web interface and tools

The homepage of the Cyanorak v2.1 information system shortly describes the origin of the genomes used to build the CLOGs database, the history of its construction and the main references that used it. The top banner available from all pages encompasses several clickable menus, including the ‘organisms page’ that lists the different genomes of the database and their characteristics, a ‘search page’ with different options to access the CLOG or gene pages of interest, a ‘BLAST scroll down menu’, a JBrowse menu giving access to direct links to the viewer of each genome, as well as several other menus providing useful information about the database (Previous Versions, References, Links, About us, Help).

#### Organisms page

**Figure 2.**
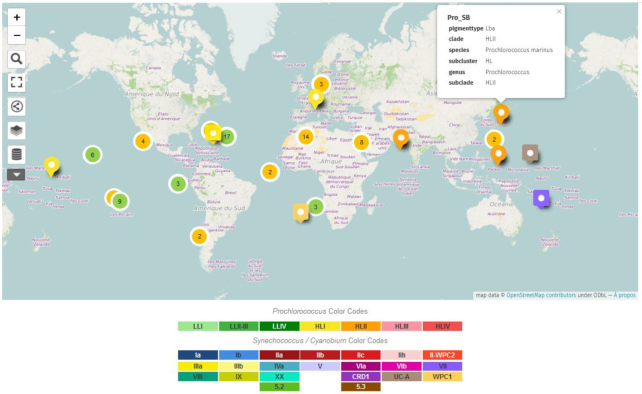
Map of the isolation sites of the different sequenced strains included in the Cyanorak v2.1 database. Green markers indicate *Prochlorococcus* strains and orange markers *Synechococcus* strains. Each marker can be expanded to reveal a ‘call-out’ that shows the strain name, pigment type and taxonomy, as shown for the North West Pacific *Prochlorococcusmarinus* SB. The Search Data boxes shown on the left hand side of the map allows to search for specific strain(s), genotype, etc.

The ‘organisms page’ consists of two tables, the first one listing *Prochlorococcus* genomes and the other one *Synechococcus* and *Cyanobium* genomes. They provide taxonomy, pigment type, sequencing center as well as various genomic characteristics (size, GC%, status, accession numbers, number of CDS, etc.) for each genome included in the database. Next to each strain name is a clickable ‘J’ logo that gives access to the JBrowse page of the corresponding genome (see below). A distribution map of all of the strains drawn with OpenStreetMap^®^ (https://www.openstreetmap.org/) is also available in this section (Figure 2), offering a flexible set of options to focus on individual strains or to select all or a subset of *Prochlorococcus* and *Synechococcus/Cyanobium* strains. Strains collected in nearby locations can also be shown with a single marker to enhance readability. In this section, each strain name in the Table can be clicked to get more detailed information (e.g. isolation site, isolator, environment ontology (ENVO) code, etc.) and also allows the user to export gene and protein sequences in FASTA format as well as whole genomes in Genbank format. In each of these files, the annotation of every gene corresponds to that found on CLOG pages (see below), which was given priority over the initial gene annotation, even if the genome was retrieved from Genbank.

#### Genomic Search tools

The main entry to the Cyanorak v.2.1 database content is through the ‘genomic search’ menu, with three possible options. The first one is a ‘quick search’ of any term mainly through ‘cluster number’, ‘gene names’ and ‘product descriptions’ fields, a term that can be searched either as an exact match or as a pattern in a more complex sentence. For instance, a search for ‘dna’ in ‘pattern’ configuration will provide a list of CDS clusters whose gene name annotation includes ‘dna’ (e.g. *dnaA*, *dnaB*, etc.) as well as a list of products whose description includes DNA (e.g. DNA gyrase, formamidopyrimidine-DNA glycolase, etc.). Search results are organized in three distinct tabs. The first one lists the matching clusters, the second one lists the matching CDS and the third one lists the matching RNA entries. Each list gives the essential information about every match and provides links to access detailed descriptions of these entries. The ‘advanced search’ option allows the user to look into any field documented in the CLOG, CDS or RNA pages, including functional categories (e.g. EC or K number, InterPro entries, GO terms, etc.) and to select one, all or a specific set of strains. Finally, the ‘phyletic pattern search’ option is used to search for CLOGs, CDS and RNA that are shared by a selection of genomes and that can be either present or absent in other strains depending on the selected option. This search option is for instance most useful to identify genes specific to a particular strain combination such as all *Prochlorococcus*, a given clade or a given pigment type.

#### CLOG page

By clicking on a Cyanorak CLOG number (format: CK_XXXXXXXX) in the result list of any search (see above), the user is sent to a cluster page providing a full description of the function and phyletic pattern of the corresponding CLOG. An indication in the upper right corner of the CLOG page specifies whether the CLOG has been manually annotated, i.e. whether an expert has edited and validated its sequence content and functional annotation. The cluster page contains several fields, including from top to bottom: (i) the corresponding gene identifier in the Cyanorak v1 database (if any), generally corresponding to the last digits of the Cyanorak v2.1 CLOG number, (ii) a gene name and its synonyms (if any) as well as the product description, (iii) functional categories including COG (5) and EggNOG (6) identifiers and their description, CyOG number (as reported in (35)), Enzyme Commission (EC) and K numbers referring to the Kyoto Encyclopedia of Genes and Genomes (KEGG) database (www.kegg.jp), ‘TIGR Roles’ and custom-designed ‘Cyanorak Roles’ (see complete description at the end of the Help menu), the latter being largely derived from ‘TIGR Roles’ but providing more details on photosynthetic and other key cyanobacterial processes, as well as gene ontology (GO) terms and their description; (iv) results of protein domain and motif searches, including TIGRFAMs, PFAMs, ProSite patterns and profiles, as well as InterPro entries, (v) numbers of related CLOGs, i.e. possible paralogs and (vi) a phyletic pattern providing the distribution of the genes in the different genomes, classified by taxonomy (genus, clade and, for *Synechococcus* SC 5.1 strains only, subclades, according to (18)), and indicating the pigment type of the corresponding strain (Supplementary Figure S2, (26,36,37)). The bottom of the page lists ORF_IDs of the different members of the CLOG, with their initial annotation, a useful piece of information when the annotation was made either automatically or by other research groups.

**Figure 3.**
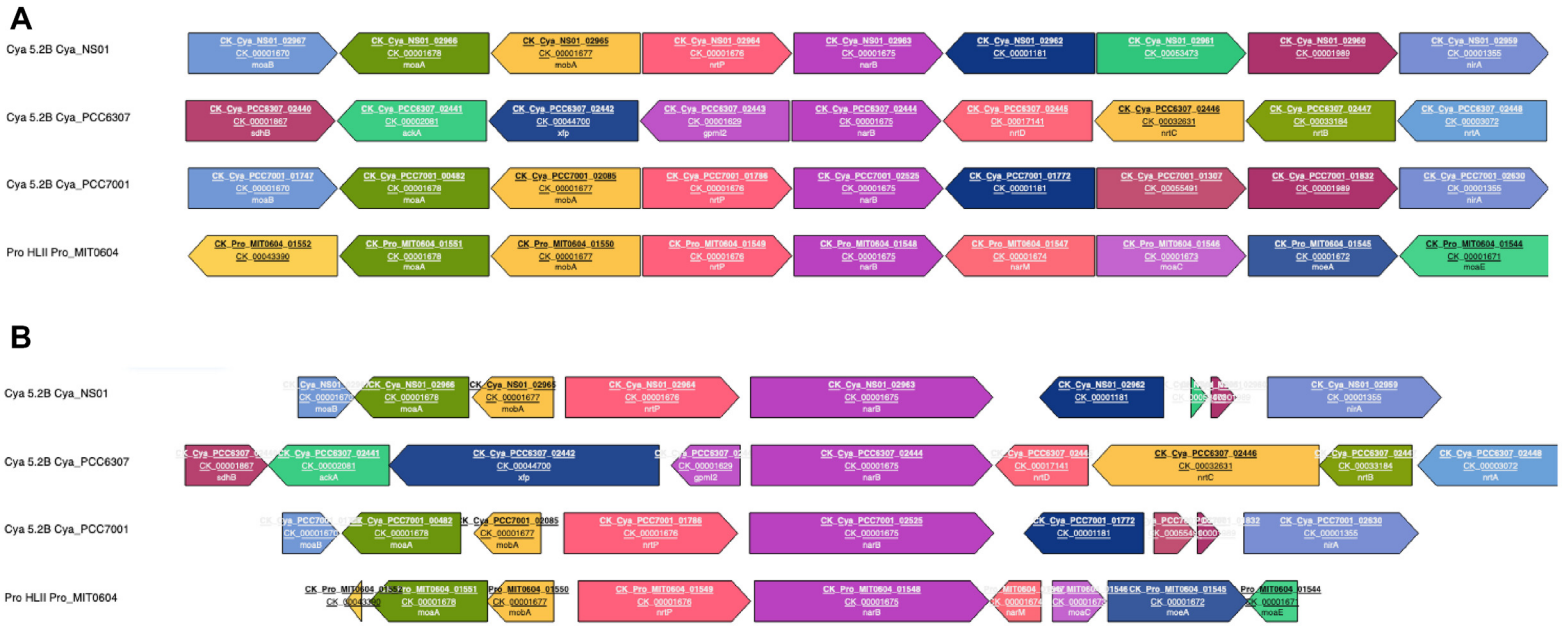
Genomic context of the *narB* gene encoding the nitrate reductase. (**A**) Genes represented at the same size. (**B**) Genes represented in relative size.

On the top left of the ‘cluster page’ is a link to the ‘genomic context’, which displays the four genes upstream and downstream of the selected gene in all members of the CLOG. Two possible representations of the context are accessible through a toggle button: genes are shown either all at the same size or in relative size (Figure 3A-B). To ease comparisons, the central gene is always represented in the forward direction whatever its original direction in the genome and the context is arranged accordingly. Each CLOG has a given (random) color and background (plain or stripped) and genome context can be regenerated around any gene of the current context by clicking on the ORF_ID of the corresponding gene, while clicking on a CLOG number opens the corresponding CLOG page.

Links at the bottom of the cluster page allow the user to export all sequences of the CLOG at once as an amino acid or nucleotide FASTA file. The descriptor of each sequence in the export is standardized and provides the genus abbreviated to the three first letters (Cya|Syn|Pro); strain name; SC, clade or subclade depending on the finest taxonomic level available for the strain as in (21); pigment type as in (36); Cyanorak ORF_ID; gene positions and strand in the genome; Cyanorak CLOG number and gene name if any (e.g. >Syn_A15–24_IIIa_3c|CK_Syn_A15–24_02629:2153016–2154431:1|CK_00000125|dnaB).

#### Gene page

By clicking on any gene in the cluster page or the relevant search result tab, the user accesses the ‘gene page’, which includes most fields previously described for the CLOG page. Specificities compared to the latter include (i) the source and location of the gene, namely the strain name, contig and gene location (position and strand) on the contig, generally consisting of the whole chromosome, (ii) a series of identifiers in Cyanorak and, if relevant, in other databases (Genbank, RefSeq, etc.) and (iii) the gene sequence in nucleotides and (for CDS) in amino acids. It must be stressed that this page contains the initial annotation of the gene (e.g. if the genome was retrieved from Genbank), and the latter often differs from the CLOG annotation which is typically much more extensive, especially if the cluster was manually curated. All genes included in Cyanorak (even when retrieved from public databases) were given, in addition to their initial ORF_ID, a unique Cyanorak ORF_ID with the standardized format CK_Genus_Strain_XXXXX (e.g. CK_Syn_PROS-U-1_00601) in order to normalize gene names and ease the identification of the genome source. Also noteworthy is that the genomes included in Cyanorak, even those that have been sequenced by other groups, have all been manually curated to some extent, including predictions of missing genes or removal of wrong predictions, and thus differ from their counterparts in other public databases (Genbank, RefSeq, etc.) not only regarding their annotation (made at CLOG level) but also their gene content.

#### BLAST

An indispensable complement to the Cyanorak database is the possibility for users to search any sequence in all genomes or proteomes of the database using two BLAST options available from a BLAST scroll down menu. This includes an implementation of the BLAST algorithm using the SequenceServer graphical interface (38) allowing to BLAST one or several nucleotide or protein sequence(s) against a selection of up to 97 genomes (‘Blast a selection’) or all genomes (‘Blast all’). Results of a BLAST search returns the Cyanorak ORF_ID, the strain taxonomy (at the SC, clades and/or subclade level) and pigment type, the CLOG number as well as the CLOG gene name and product (e.g. CK_Syn_A15–24_00652_III_IIIa_3c CK_00001060!rpoC1!DNA-directed RNA polymerase complex, gamma subunit).

#### JBrowse page

**Figure 4.**
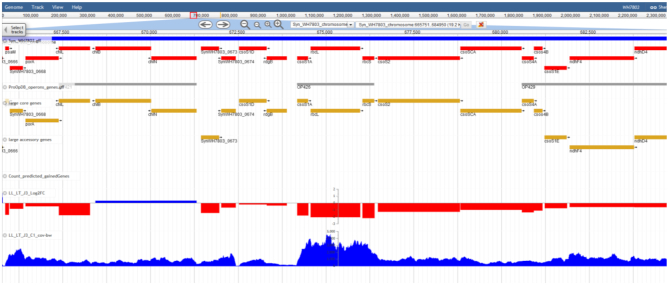
An example of genome visualization using the JBrowse plugin of Cyanorak v2.1.

Clicking on the ‘J’ logo next to each strain name in the JBrowse page (or ‘Organisms’ page) gives access to a JBrowse viewer (39) allowing the user to visualize the whole annotated genome and to zoom in to see the local context and detailed annotation of any gene, as derived from the ‘CLOG page’ (see above, Figure 4). Left clicking on a gene gives access to the detailed functional annotation of the corresponding gene, with hyperlinks to Cyanorak and external functional databases. The genes can be searched by annotation or Cyanorak ORF_ID. A ‘select tracks’ menu gives access to additional data associated with each genome, when available. These include strict and large core and accessory genomes, gained genes as well as genomic islands, as determined in a recent comparative genomic study of 81 non-redundant picocyanobacteria genomes (9). Also available are operon predictions using ProOpDB (40) and transcriptomic data. The latter can be visualized by bibliography, experiment, acclimation condition (e.g. low or high light) or stress conditions (e.g. exposure to low temperature or UV radiation). In the current version of Cyanorak, transcriptomic data are available for *Synechococcus* sp. WH7803 (29,34) and a number of other *Prochlorococcus* or *Synechococcus* strains studied by other groups, but in this case expression data are only displayed as log2(Fold Change).

#### Exports

Various exports are available from different pages of the Cyanorak v2.1 information system, including strain and genome characteristics from the organism page, annotated complete genomes from the individual strain pages, individual fasta protein and nucleotide sequences from gene pages and multifasta protein and nucleotide sequences from cluster pages.

## DISCUSSION

As the most ancient photosynthetic organisms, cyanobacteria had a key role in the oxidation of the primitive Earth atmosphere (41) but also in the primary endosymbiosis, an event that led to the advent of green and red algae and ultimately to all eukaryotic oxygenic phototrophs (42). Besides their relevance in evolutionary biology, cyanobacteria are also of great interest in ecology, given their ubiquity and abundance in many ecosystems, including oceans and deserts (43) and the noxious impacts of their bloom-forming toxic representatives in freshwater environments (44). For all these reasons, but also thanks to their fairly small genomes sizes, ranging from 1.4 to 11.6 Mb, these microorganisms have been the subject of many sequencing projects (8,9,12,45), which in turn triggered the generation of a number of dedicated genome databases. The oldest of these databases is Cyanobase (http://genome.microbedb.jp/CyanoBase), initially created to provide access to the first sequenced cyanobacterial genome, the model freshwater strain *Synechocystis* sp. PCC6803 (46,47). This database has since then been extended to host many more recently sequenced freshwater and marine cyanobacterial genomes (376 entries, including 86 complete genomes in April 2019), but has been ‘under maintenance’ since summer 2019. Although this database provides much useful genomic information, this is not a CLOG database and is therefore not designed to make extensive genomic comparisons. Also worth noting, CyanoClust (http://gclust.c.u-tokyo.ac.jp/CyanoClust/) is a database of homologous groups initially limited to cyanobacteria and plastids and which was more recently extended to heterotrophic bacteria and Archaea (48). It provides lists of orthologs generated by the program Gclust, but functional annotation is limited to the original product description and fasta sequence of individual members of each CLOG. The database that was most similar to Cyanorak v2.1, was the ‘*Prochlorococcus* portal’ a.k.a. ‘Proportal’ (49). It was also based on CLOGs computed from a number of *Prochlorococcus* and marine *Synechococcus* genomes, with a strong focus on the former genus. Since 2018, this database has however been merged to the Joint Genome Institute Integrated Microbial Genomes and Microbiomes (JGI-IMG) and renamed ‘IMG-Proportal’ (http://img.jgi.doe.gov/cgi-bin/proportal/main.cgi). The latter site lists all publicly available genomic, transcriptomic, metagenomic and population data on *Prochlorococcus*, *Synechococcus* and their cyanophages, which can be analyzed using the IMG’s data warehouse and comparative analysis tools (50), but the initial CLOG-centered organization of Proportal has been lost.

Compared to these databases, Cyanorak is more function-oriented and aims to provide rich and up-to-date functional annotations of CLOGs, with a preference for those derived from genes or proteins that were characterized in cyanobacteria. In contrast to most large CLOG databases currently available, such as COG (5) or EggNOG (6) that encompass very distantly related organisms, Cyanorak is focused on *Prochlorococcus* and marine *Synechococcus/Cyanobium*, i.e. a monophyletic and homogenous group of microorganisms sharing a similar morphology and lifestyle, making more reliable the assumption that reciprocal best hits in different genomes truly correspond to orthologs. Thanks to this CLOG-based approach, the continuous expert curation efforts employed since the mid-2000’s have allowed us to improve the annotation of all genomes of the Cyanorak database, even those initially retrieved from Genbank. Furthermore, a number of genes that were missing in these often automatically annotated genomes have been added, while many ORF over-predictions have been suppressed, so that both gene content and annotations differ between genomes in Cyanorak and their counterparts in large public databases.

Another important asset of Cyanorak is the variety of tools for exploring and exporting genomes from the database. For instance, one can search for CLOGs common or specific to a particular phylogenetic group of interest, an approach that can provide clues to identify genes encoding a specific function. For instance, searching Cyanorak for homologs of MpeZ, an enzyme involved in type IV chromatic acclimation (CA4), i.e. a reversible pigmentation change occurring in some *Synechococcus* strains when shifted from blue to green light (26), allowed us to identify a second type of chromatic acclimation, so-called CA4-B, which possess MpeW, a MpeZ homolog. Both the *mpeZ* and *mpeW* genes are located in a specific genomic island, but gene content, organization and genomic context differ between the CA4-A and CA4-B islands. Another interesting example concerns the chlorophyll (Chl) biosynthesis pathway. It is well known that *Prochlorococcus* lacks monovinyl-Chl *a*, which is replaced by divinyl-Chl *a*, even in reaction centers (51,52). Comparing the genomic context of the core *malQ* gene (encoding a glucanotransferase) between all genomes of the Cyanorak database shows that in marine *Synechococcus* this gene is always preceded by an enzyme that reduces divinyl-Chl *a* into mononyl-Chl *a*, but surprisingly there are two possible mutually exclusive reductase genes depending on strains, either *dvr* (53) in strains from clades I-IV, VII, CRD1, WPC1 XX and UC-A or *cvrA* (54) in all other *Synechococcus/Cyanobium* lineages (Supplementary Figure S3). Dvr and CvrA possess the same enzymatic function but share no sequence identity, and are thus analogs. In *Prochlorococcus* genomes, neither *dvr* nor *cvrA* are found upstream of *malQ*, and none of these genes is found elsewhere in the genome, explaining why these strains are all incapable of producing monovinyl-Chl *a*.

Cyanorak v2.1 is also a repository for a variety of transcriptomic data, the interpretation of which relies heavily on the quality of genome annotation. In Cyanorak, these data are connected to the genome database through a JBrowse interface, which also gives access to genomic features such as predicted operons, gained genes or the core or accessory nature of genes, which can be used to further refine the interpretation of gene expression data (see e.g. (34)).

## FUTURE DEVELOPMENTS

The current version of the database includes rRNAs, tRNAs and tmRNAs, but still no small RNAs (sRNAs), so we plan to add such information in a forthcoming release, at least for the most conserved sRNAs. Another planned improvement of the database concerns the curation of gene starts. Although many gene starts have been corrected manually, amino acid alignments readily made from exports of CLOG pages show that a large number of those starts are still mis-predicted, leading to seemingly too short or too long sequences in a number of genomes. We will thus develop an application that allows to automatically correct likely wrong starts, at least when N-termini are not too variable. Finally, new fields will be added on the CLOG page, including for instance orthologs of each CLOG in relevant biological models, such as the freshwater cyanobacteria *Synechocystis* sp. PCC 6803 and *Synechococcus* sp. PCC 7942, the heterotrophic bacteria *Escherichia coli* and *Bacillus subtilis* or the higher plant *Arabidopsis thaliana*.

Future versions of the Cyanorak database will include genomes newly released from public data as well as a number of transcriptomes either generated by our team or by external users, providing that they follow instructions described in the Cyanorak help file. It must be stressed that the aim of Cyanorak is not to host all of the rapidly-growing number of incomplete SAGs and MAGs, apart from a few representative uncultivated lineages (e.g. *Synechococcus* EnvA/B, *Prochlorococcus* HLIII-VI). Thus, a pipeline is currently being developed to easily transfer the rich functional annotation of the Cyanorak genomes to these new partial genomic sequences and, more generally, to any picocyanobacterial environmental reads retrieved from metagenomes and metatranscriptomes. A few previous studies, where annotations made in Cyanorak were used to analyze omic data, have notably allowed us to (i) compare the nitrogen assimilation capacities of *Prochlorococcus* populations from inside and outside the Agulhas rings in the South Atlantic Ocean (55), (ii) highlight differences between *Prochlorococcus* and *Synechococcus* populations in their adaptation and acclimation responses to iron deficiency in the vicinity of the Marquesas island (56) and (iii) demonstrate through the global oceanic distribution of desaturase genes the key role of these enzymes involved in the modulation of membrane fluidity for the colonization of different thermal niches by distinct *Synechococcus* lineages (28). We envision that Cyanorak will become a reference genome database for the taxonomic and functional annotation of not only newly released genomes and transcriptomes of marine picocyanobacteria, but also the ever-increasing marine meta-omes, which given the natural abundance and ubiquity of these microorganisms in the marine environment constitute a significant part of all reads retrieved from the upper lit layer of marine waters.

## DATA AVAILABILITY

The Cyanorak v2.1 information system is available at http://www.sb-roscoff.fr/cyanorak.

## Supplementary Material

gkaa958_Supplemental_File

## Notes

Present address: Martin Ostrowski, Climate Change Cluster, University of Technology, Broadway, NSW 2007, Australia.

Present address: Hugo Doré, Institute for Collaborative Biotechnologies and Department of Ecology, Evolution, and Marine Biology, University of California, Santa Barbara, Santa Barbara, California, USA.
