# Supplementary material for: Cyanorak v2.1: a scalable information system dedicated to the visualization and expert curation of marine and brackish picocyanobacteria genomes
Source: Nucleic Acids Res. 2020 Oct 30;49(D1):D667–76. doi: 10.1093/nar/gkaa958 (PMC7779031; doi:10.1093/nar/gkaa958)
Supplement: gkaa958_Supplemental_File [file gkaa958_supplemental_file.pdf]

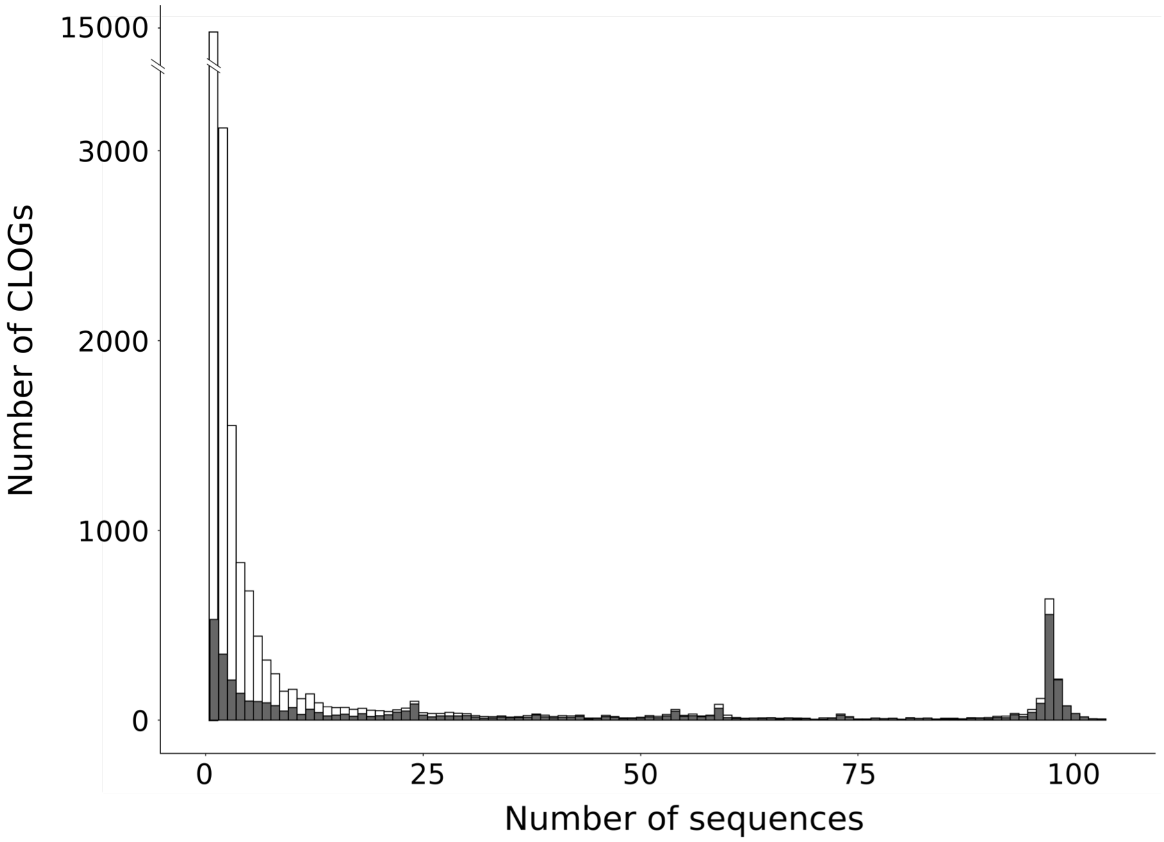

Figure S1. Distribution of the number of sequences per CLOG. The fraction of CLOGs that have been manually curated is shown in dark grey. Note that 21 CLOGs gathering more than 105 genes per CLOG, all manually curated, are not shown on the figure.

Phyletic Pattern

Genus	Group	Clade	Strain	Pigment type	Copies
Prochlorococcus	HL	HLI	EQPAC1	Lba	0
			MED4	Lba	0
			MIT9515	Lba	0
		HLII	AS9601	Lba	0
			GP2	Lba	0
			MIT0604	Lba	0
			MIT9107	Lba	0
			MIT9116	Lba	0
			MIT9123	Lba	0
			MIT9201	Lba	0
			MIT9202	Lba	0
			MIT9215	Lba	0
			MIT9301	Lba	0
			MIT9302	Lba	0
			MIT9311	Lba	0
			MIT9312	Lba	0
			MIT9314	Lba	0
			MIT9321	Lba	0
			MIT9322	Lba	0
			MIT9401	Lba	0
			SB	Lba	0
			UH18301	Lba	0
		HLIII	HNLC2	Lba	0
		HLIV	HNLC1	Lba	0
	LL	LLI	MIT0801	Hba	0
			NATL1A	Hba	0
			NATL2A	Hba	0
			PAC1	Hba	0
		LLII	LG	Hba	0
			MIT0602	Hba	0
			MIT0603	Hba	0
			SS120	Hba	0
			SS2	Hba	0
			SS35	Hba	0
			SS51	Hba	0
			SS52	Hba	0
		LLIII	MIT0601	Hba	0
			MIT9211	Hba	0
		LLIV	MIT0701	Hba	0
			MIT0702	Hba	0
			MIT0703	Hba	0
			MIT9303	Hba	0
			MIT9313	Hba	0

Genus	Subcluster	Clade	Strain	Pigment type	Copies
Synechococcus/Cyanobium	Subcluster 5.1	Ia	CC9311	3dA	3
			WH8020	3dA	0
		Ib	MVIR-18-1	3aA	4
			PROS-9-1	3dA	3
			ROS8604	3a	6
			SYN20	3a	4
			WH8016	3aA	4
		IIa	A15-44	2	2
			M16.1	3a	2
			RS9902	3c	2
			RS9907	3a	3
			TAK9802	3a	2
			WH8109	3bB	2
		II-WPC	KORDI-52	3bB	2
		IIc	A15-62	3dB	2
			CC9605	3c	2
		IIh	PROS-U-1	3dB	3
		IIIa	A15-24	3c	2
			A18-40	3dB	2
			A18-46.1	3c	3
			BOUM118	3c	2
			RS9915	3dB	3
			WH8102	3c	3
			WH8103	3bB	2
		IIIb	A15-28	3c	2
		IVa	BL107	3dA	4
			CC9902	3dA	3

Genus	Subcluster	Clade	Strain	Pigment type	Copies
Synechococcus/Cyanobium	Subcluster 5.1	V	BMK-MC-1	2	3
			WH7803	3a	3
		VIa	MEDNS5	3c	3
			WH7805	2	5
		VIb	PROS-7-1	2	3
		VIIa	A15-60	3c	2
			A18-25c	3c	2
		VIIb	NOUM97013	3a	2
		VIII	RS9909	1	3
			RS9917	1	3
			WH8101	1	3
		IX	RS9916	3dA	4
		CRD1	BIOS-U3-1	3dA	5
			MITS9220	3dA	2
			BIOS-E4-1	3cA	3
		WPC1	A15-127	3c	2
			KORDI-49	3aA	2
		XX	CC9616	3c'	1
		UC-A	KORDI-100	3c'	2
	Subcluster 5.2	5.2	CB0101	1	1
			CB0205	2	0
			NS01	1	3
			PCC6307	1	2
			PCC7001	1	3
			WH5701	1	3
	Subcluster 5.3	5.3	MINOS11	3dB	3
			RCC307	3eA	3

Figure S2. Example of phyletic pattern for the *psbA* gene copies encoding the D1.2 isoform of the D1 protein of photosystem II. Note that this D1 isoform is absent from all *Prochlorococcus* strains, while it can be present in up to 6 copies in *Synechococcus/Cyanobium* strains.

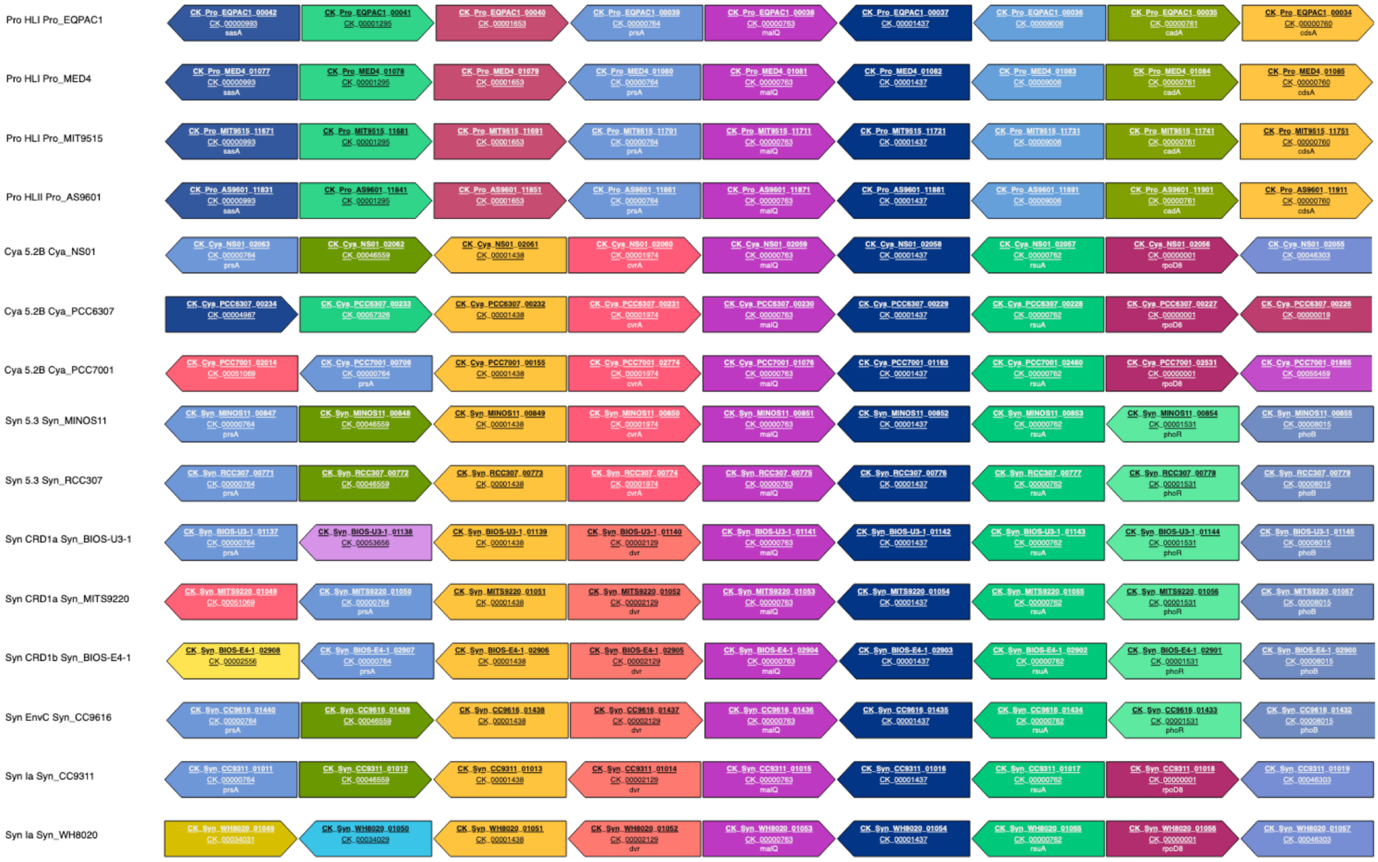

Figure S3: Example of the genomic context of the *malQ* gene encoding the 4-alpha-glucanotransferase. This example shows that *Synechococcus* strains possess either *cvrA* or *dvr* upstream the *malQ* gene, while *Prochlorococcus* strains possess neither of these genes (see text for explanations).
